# Continuous training based on the needs of operating room nurses using web application: a new approach to improve their knowledge

**DOI:** 10.1186/s12909-024-05315-3

**Published:** 2024-03-26

**Authors:** R. Khorammakan, S. H. Roudbari, A. Omid, V. S. Anoosheh, A. N. Arabkhazaei, A. Z. Arabkhazaei, J. Khalili, H. Belyad Chaldashti, A. Ghadami

**Affiliations:** 1 Department of the Operating Room, School of Nursing and Midwifery, Hormozgan University of Medical Sciences, Bandar Abbas, Iran; 2 Department of the operating room, Farmaniyeh hospital, Tehran, Iran; 3 Department of Medical Education, Medical Education Research Center, Isfahan University of Medical Sciences, Isfahan, Iran; 4 Department of Occupational Health and Ergonomics, Student Research Committee, School of Health, Shiraz University of Medical Sciences, Shiraz, Iran; 5 Department of Operating Room, Torbatjam Faculty of Medical Sciences, Torbatjam, Iran; 6 Department of Operating Room, School of Paramedical Science, Gonabad University of Medical Sciences, Gonabad, Iran; 7 Ansar Al-Ghadir Hospital, Shahid Beheshti University of Medical Sciences, Tehran, Iran; 8 Department of Operating Room, Shahid Ansari Hospital, Rudsar, Iran; 9 Department of the Operating Room, Nursing and Midwifery Care Research Centre, School of Nursing and Midwifery, Isfahan University of Medical Sciences, Isfahan, Iran

**Keywords:** Continuous education, Knowledge, Satisfaction, Operating room nurse

## Abstract

**Introduction:**

Since university education and intensive and limited pre-service training do not provide an acceptable level of performing the duties of operating room nurses, and considering the limitations of traditional training methods in the field of operating room; This study was conducted with the aim of determining the effect of using the electronic education approach based on web application, leveled, personalized and based on the needs of nurses on their level of knowledge and satisfaction.

**Materials and methods:**

This research is a quasi-experimental type of single-group multi-center pre-test-post-test, which during that, four stages of educational needs assessment, educational content design, web application design for training and evaluation of operating room nurses and determining the effectiveness of this method are included. Based on their knowledge and satisfaction, during this period, 36 nurses from the operating rooms that met the study criteria were included in the study by stratified random sampling based on the determined sample size. The data collection includes a four-choice test to measure the knowledge of operating room nurses in heart anatomy (score range 0–20), the principles of movement, transferring and positioning of the patient in the operating room (score range 0–15), the principles of ergonomics in the operating room (score range score 0–10) and satisfaction questionnaire (score range 0–28). Data collected using descriptive statistical tests (percentage of frequency and frequency, mean and standard deviation) and analytical tests (paired sample t-test, independent samples t-test, ANOVA, Pearson correlation, chi-square) with the software SPSS version 16 was analyzed.

**Results:**

Generally, the average knowledge scores of operating room nurses before and after the intervention were 5.96 ± 3.96 vs. 13.6 ± 3.77, in the course of principles of moving, transferring and positioning the patient in the operating room were 6.3 ± 3.42 vs. 13.3 ± 1.32, respectively 8.7 ± 3.97 vs. 18.1 ± 1.07 (in heart anatomy), 1.57 ± 2.6 vs. 0.73 ± 9.1 (in the principles of ergonomics in the operating room) and the average Knowledge scores after the intervention were significantly higher than before the intervention (*P*<0.001). Also, the average satisfaction score of nurses was 21.3 ± 5.83 and 22 nurses (64.7%) were satisfied with the e-learning course.

**Conclusion:**

The use of the electronic education approach based on the web application, leveled, personalized and based on the needs of the nurses, led to the improvement of the level of knowledge and satisfaction of the operating room nurses. E-learning can be used as a complementary educational tool and method for continuous training of operating room nurses in other specialized fields of operating room and surgery.

**Highlights:**

• Educational content in the form of educational videos taught by professors of medical sciences universities on each of the topics of heart anatomy (28 episodes of 5–10 minutes), principles of ergonomics in the operating room (7 episodes of 5–25 minutes) and movement principles. The transfer and positioning of the patient in the operating room (16 episodes of 10–20 minutes) were designed in three primary, intermediate and advanced levels.

• The results of this study showed that the use of an electronic education approach based on the web application, levelled, personalized and based on the needs of nurses, led to the improvement of the knowledge of operating room nurses. Also, operating room nurses were delighted with electronic training courses. E-learning can be used as a complementary educational tool and method for continuous training of operating room nurses in other specialized fields of operating room and surgery.

• Based on the results of this study, the use of an electronic education approach based on the needs of operating room nurses can be used as a complementary tool to conventional continuous education. Since this method allows interactive, personalized education is levelled, and asynchronous. It can be used at any time and place on a laptop, tablet or mobile phone; a wide range of operating room nurses in the hospitals of the Islamic Republic of Iran can use it for educational justice to Many borders should be established in the country. However, there are studies to evaluate the generalizability and the effect of using the e-learning approach on the clinical skills of operating room nurses and to compare the effect of e-learning with other methods and educational tools on the knowledge and skills of the learners and the extent of consolidating the learned material in their memory.

## Introduction

The operating room is a very complex environment and system where the caregiver, the patient and the technology are gathered in a physical environment in order to achieve the desired results for the patients [1, 2]. In today’s complexity and transformation of the world, the continuity and existence of organizations depends on creating a balance between the development of human resources, methods and technologies in organizations and adapting to changes and departmental innovations. According to the World Health Organization, the performance of any system depends on a combination of the skills, availability and performance of its human resources, and the scientific and practical abilities of personnel in various fields on their own safety and patients’ safety, as well as providing the best services in the direction of treatment. It has a direct and significant effect on patients [3, 4]. Therefore, most of the advanced countries of the world have realized the importance of human resources as a part of vital and strategic resources and productive assets, and in order to strengthen their knowledge, skills and abilities, they prepare and implement various programs.

Today, one of the basic measures that leads to the efficiency of organizations is the creation or acquisition and continuous development of human resource through the implementation of training and improvement programs, which at the individual level increases the value of the individual, and at the organizational level, improves and develops the organization, and at the national and even transnational level, it leads to an increase in productivity [5, 6]. Since official academic education and intensive and limited pre-service training do not sufficiently and acceptably prepare hospital staff to perform their duties in the clinical environment, the implementation of the training program has become more necessary [7]. Due to the fact that each person has unique characteristics, the way of learning skills and learning needs are also different, so the first and most basic step in education is to examine educational needs.

Examining educational needs is a process during which needs are identified and planned and acted upon according to priority [8, 9] and it is considered as a basis for preparing special educational content and a basis for setting goals and thus providing a suitable platform for organizing other important elements around prioritized needs; By preventing rework, it ultimately leads to increasing the effectiveness and efficiency of human resources, reducing waste, developing knowledge, skills, increasing job satisfaction and motivating employees [5, 8, 10–19]. In the study of Qalaei et al. (2013), which was conducted with the aim of determining the effectiveness of in-service training courses for nurses in medical centers affiliated to the Social Security Organization, the results showed that the lack of correct needs assessment caused the lack of overlap between training programs and the training needs of nurses [7]. In the study of Mazoji et al. (2015), the results showed that nurses who work in eye surgery operating rooms need training and informing and refresher courses about drug information, the nature of surgery and especially eye surgery techniques [20].

The most widespread method of continuous training of medical personnel is the face-to-face training method, which many studies have shown that this traditional training method has many limitations, such as not recognizing the needs of learners and their personal differences, and not addressing high-level cognitive skills such as problem solving and creative thinking [21]. As a result, many researchers have emphasized that traditional educational methods need to be changed and modified by modern educational methods [21, 22]. Electronic education has been proposed as one of the complementary educational methods [21, 23–26] and researchers have come to the conclusion that to modify the time and place limitations associated with traditional education, the possibility of lifelong learning and appropriate to the specific conditions of each individual, e-learning increasingly provides easy access to education and can be a suitable complement to traditional education [21, 27–43]. In the field of the operating room, e-learning is also possible due to the possibility of providing cost-effective training, at any time and place, without worrying about endangering the patient’s safety, sharing educational materials, facilitating the updating of educational content, using different learning styles according to It has become popular with the needs and abilities of each learner and the adjustment of learning speed by each learner according to his characteristics [21, 44–53]. Various studies have shown that the use of electronic education methods in the nursing profession has led to an increase in the satisfaction and professional progress of nurses in hospitals [54].

Since academic education and intensive and limited pre-service trainings do not provide an acceptable level of effective performance of duties by operating room nurses, and also considering the limitations of the traditional training method in the operating room field; Therefore, the necessity of planning to prepare, formulate and implement electronic training courses related to individual, occupational and organizational needs as a means of informing and responding to rapid changes in the health system as well as improving professional knowledge and skills is felt more. This study sought to answer the question that to what extent the training of specialized topics of the operating room profession, electronic education based on web application, leveled, asynchronous, personalized and based on the needs of nurses can lead to the promotion of specialized knowledge and Will operating room nurses be satisfied with the new teaching method?

## Materials and methods

### Study design

This study was done as a semi-experimental single-group, multi-center pre-test, post-test in four phases of educational needs assessment, educational content design, web application design for training and evaluation of operating room nurses and determining the effect of operating room nurse training based on the web application on the level of knowledge and satisfaction.

### Ethical considerations

First, the code of ethics was obtained from the regional committee of ethics in medical science research. Then the process of conducting the study and its objectives were explained by the researcher through WhatsApp messenger to each of the operating room nurses who met the criteria for entering the study, and then the online informed consent form (in WORD file format) was completed by each of them and it was delivered to the researcher through WhatsApp messenger in the form of a WORD file.

### Sampling method and sample size

To evaluate the effect of this study, at least 29 volunteer operating room nurses was needed (according to formula 1), and based on the results obtained from similar study [55] and estimated losses(20%), a sample of this size would allow us to detect a somewhat large effect size, on the order of 2.62(d), with a confidence interval of 95%(z1) and power of 80%(z2), with a standard deviation of 3.62 score for pre-test(s1) and standard deviation of 3.49 score for post-test, this sample size would allow us to find mean differences of 1.57 scores.

## Study phases

### Training needs assessment

At this stage, according to our previous study [56], which aims to determine the need for training and improving the knowledge of operating room nurses in Al-Zahra, Amin, Kashani and Chamran hospitals in Isfahan, Iran, in the areas of general and specialized knowledge of the operating room and the need for counseling to improve motivation and their job and the level of need to launch a web application with the purpose of special training for operating room nurses was done in an organized and leveled manner, and the results showed that operating room nurses in the field of general and specialized knowledge and heart anatomy topics (95%, 38 people), the principles of ergonomics in the operating room (95%, 38 people) and the principles of moving, transferring and positioning the patient in the operating room (90%, 36 people) as the sub-fields of this field, need the most training and knowledge improvement; Therefore, in the second phase, we designed educational content to train operating room nurses in the mentioned topics.

### Educational content design

**Fig. 1:**
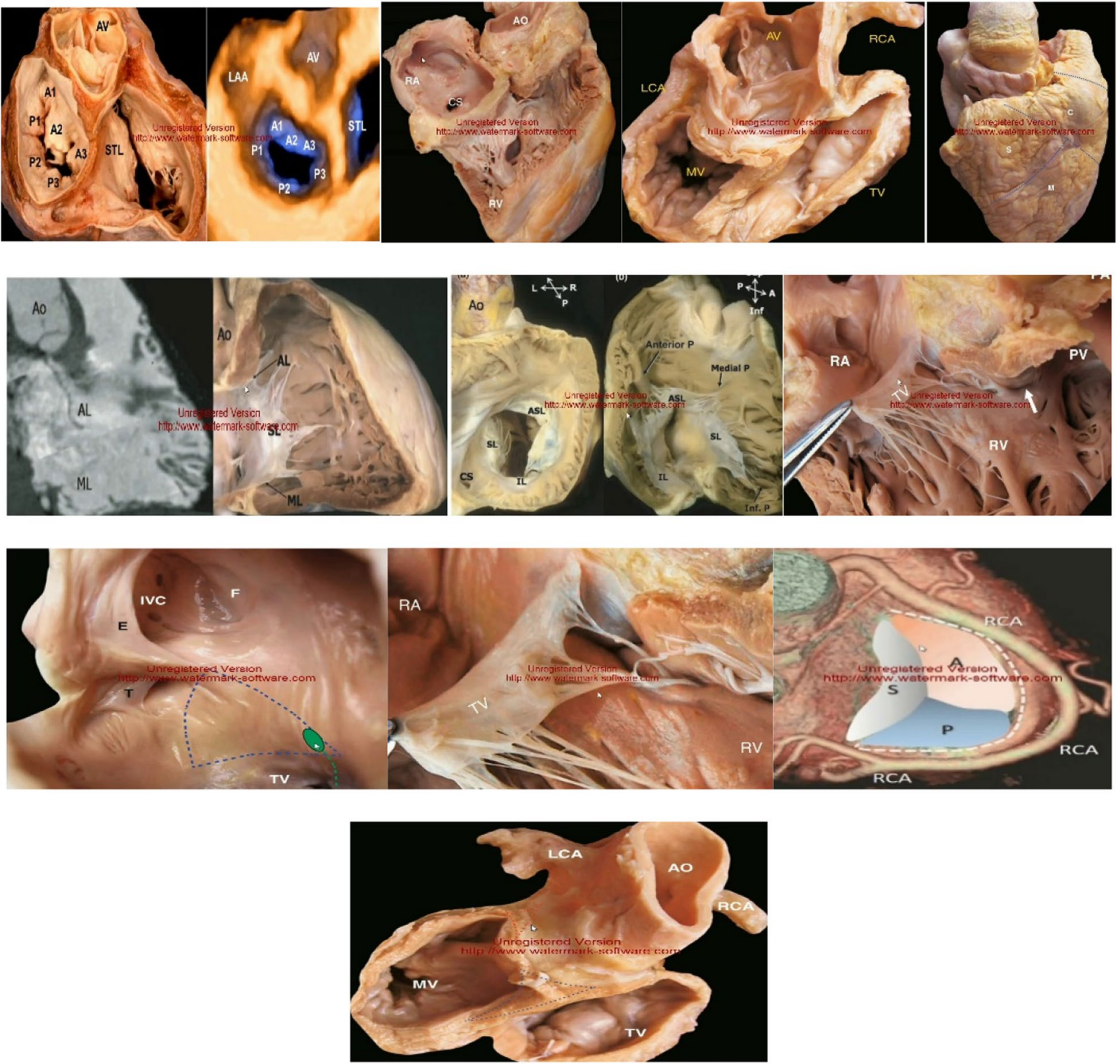
Educational content of heart anatomy: Tricuspid valve anatomy

At first, for each training course, a panel of 10 experts consisting of professors from the surgical technology department of the University of Medical Sciences (with at least 5 years of experience in teaching theoretical and clinical courses in surgical technology and at least a bachelor’s degree in surgical technology), operating room nurses working in Hospitals with at least 10 years of experience in the operating room, hospital operating room supervisors with at least 5 years of experience in the operating room, cardiac surgeons with at least 15 years of experience in heart surgery, professors of the Medical Education Department of the University of Medical Sciences, professors of the Ergonomics Department with at least 5 years of teaching experience in the field of ergonomics and at least a master’s degree in ergonomics), was formed and during 3 sessions, the theoretical and clinical educational needs of operating room nurses, the problems in the training of operating room nurses and the results of phase 1 were examined.4, was discussed and based on the results of the expert panel meetings and also by using authentic books in the fields of cardiac anatomy, ergonomics and the principles of moving, transferring and positioning the patient in the operating room [57–72], the research team with the help of professors to Designed the educational content of each course. In the next step, the educational content in the form of educational videos was provided to the panel of experts for content validation, and their opinions regarding the adaptation of the educational content to the needs of operating room nurses were obtained, and the validity of the educational content of each course was approved by all members. The panel of experts (10 people) arrived. Among the opinions of the expert panel members regarding the educational content, it is possible to improve the quality of the teachers’ voices, use a white background to display the educational content, use more images instead of text, and reduce the duration of each video to a maximum of 20 minutes in order to avoid the fatigue of the learners in the virtual direction. Finally, educational content in the form of educational videos taught by professors of medical sciences universities in each of the topics of heart anatomy (28 episodes of 10 5 minutes), principles of ergonomics in the operating room (7 episodes of 25 5 minutes) and principles of movement, transferring and Patient positioning in the operating room (16 episodes of 20 10 minutes) was designed in three levels: basic, intermediate and advanced (Fig. 1).

### Web application design for training and evaluation of operating room nurses

At first, an expert team with 3 years of experience in designing, programming and building quality web applications was added to the research team. This team designed the initial and experimental version of the web application during 3 months and the initial version was given to the panel of experts who were also present in phase 4 and 2 to check the validity of the content, and the panel members reviewed all the panels of the web application during 4 sessions. Among the comments of the expert panel members, it is possible to perform the initial registration of users by the admin (creating a username and password for each user), creating and maintaining the security of virtual tests in all tests of each course, adding the learning objectives of each course after completing the test. The level pointed out that it was applied in the web application and its edited version was again provided to the members of the panel of experts and was approved by all the members of the panel.

The application was designed on the web and contains four panels of training courses in each of the subjects of heart anatomy, principles of ergonomics in the operating room and principles of moving, transferring and positioning the patient in the operating room, discussion forum, about us and contact us, and how Registration and comprehensive use of this application is as follows:

After entering the application link in the browser and using a mobile phone, tablet or laptop, the user will face the application login page. On this page, if the user has registered in the application, he can enter the application space by entering the username and password and clicking on the login option. Also, if the user has forgotten his password, he can recover his password by clicking on the forgotten password option. New users should complete the registration form (demographic information questionnaire) by selecting the registration option and entering the username and password created by the admin. After entering the application, the user will face a page that should choose the topic among the three topics of heart anatomy, principles of ergonomics in the operating room, and principles of moving, transferring and positioning the patient in the operating room. By choosing a subject, the user will enter a page where the user will be warned that if wrong topic was chosen, he/she can change it by choosing the option “change the educational topic”, and if the option “enter the test page” is clicked, the user enters the relevant level test page and reads the rules of participating in the test, and by clicking on the test start option, the questions (in the form of four-choice questions) will be displayed for him, and it will not be possible to change the educational topic.

It should be noted that the following measures have been taken to create and maintain the security of all tests:

- Questions do not have numbers.
- The options for each question do not have numbers.
- It is not possible to copy questions and options.
- The time to answer each question is about 20 seconds.
- The order of displaying questions and the options of each question is random and is different for each user
- Correct answers will not be shown to the users during or at the end of the test
- Access to the educational content of each course and topic is limited during the exam

After completing the level determination test, the user’s score and his level (very poor, poor, average and excellent) will be displayed and by clicking on the training panel option, the user will enter the training course according to his level and will get acquainted with the course instructor and the educational topics of different levels (basic, intermediate and advanced). Each user will have one of the following four statuses at the end of the level determination test in which of the topics:

1. A user who is at a very poor level in the placement test will enter the basic level training course of his chosen subject and will have 14 days to study the training content of his basic level course and on the fourteenth day (in order to reduce errors Reminder [73–78] The final exam of the basic level course (four-choice exam) has been activated for him and the user will participate in the exam (within a period of 14 days, at least 2 times using the communication method that the user has chosen in his registration form, including WhatsApp, email, Telegram, he will be reminded of the exam time) and if the user receives a passing score, the intermediate level training course of his chosen subject will be available and, like the basic level course, the user will have 14 days to study the contents and on the 14th day will participate in the final exam of the intermediate level course, and if the exam is passed successfully, the advanced level training course of the subject would be available, and the user will have 14 days to study advanced level educational content and participate in the final exam of the chosen subject on the 14th day. In case of passing the advanced level exam, the user will enter the satisfaction questionnaire page and after completing the form, a course completion certificate will be awarded.
2. A user who is at a weak level in the placement test will enter the intermediate level training course of his chosen subject and will have 14 days to study the contents and participate in the end of the intermediate level course on the 14th day. and if a passing grade is accomplished, the user will enter the advanced level training course of the chosen subject and will have 14 days to study the advanced level educational content and on the fourteenth day will participate in the final exam of the chosen subject and if a passing grade is accomplished, the user will enter the questionnaire page After completing the questionnaire, a certificate of completion will be given. It should be noted that if desired, the user can have access to the basic level educational content of the selected subject through the option of ‘training courses’ in the user panel section and participate in the final exam of the basic level course.
3. A user who is placed at the intermediate level in the placement test, enters the advanced level training course of the selected topic and will have 14 days to study the advanced level educational content and participate in the final test of the selected topic on the 14th day. If a passing grade is accomplished, the user will enter the satisfaction questionnaire page and after completing the questionnaire, a course completion certificate will be awarded. It should be noted that if the user wishes, can have access to the educational content of the basic and intermediate level of the chosen subject and participate in the end-of-course exam.
4. A user who is at an excellent level means that it is not necessary to participate in the training courses designed by our team and the educational content of the mentioned system cannot lead to the improvement of his knowledge; However, if the user wishes, can have access to the educational content of all levels of the chosen subject and participate in the end-of-course exam of each level and the final exam.

In the user panel section, which is displayed and accessible to the user after completing the placement test, the user will be faced with options to contact us, about us, discussion forum, my training panel, training courses and exit.

On the ‘contact us’ page, there are ways to communicate with the application admin and the study team through which any user can communicate with us and raise their questions and problems in the field of using the application.

On the ‘About Us’ page, the user gets acquainted with the goals, vision and mission of our team in web application, design, and the training courses available in it, the web application design team and its supporters.

On the forum page, the user can exchange opinions and discuss with the instructor of the selected course and other users of the same level in the training course of the selected subject through text messages.

The user can be quickly transferred to the page of his educational content by clicking on the ‘My Education Panel’ option.

### Determining the effectiveness of the training of operating room nurses based on the web application on their knowledge and satisfaction

At this stage, 36 operating room nurses working in four selected hospitals in Isfahan, Iran, who meet the criteria for entering the study, were selected by stratified random sampling method and after a full explanation of the study process and its objectives by the researcher through message, the study was started by sending WhatsApp and completing the online informed consent form (in WORD file format). The criteria for entering the study include a history of at least 3 months of working in the operating room, being employed in the operating room of one of the studied hospitals, having consent to participate in the study, having at least a academic education with an associate degree, having a computer, tablet or a mobile phone with the ability to connect to the Internet to enter the web application, having the internet with a suitable speed to enter the web application, and the exit criteria also include unwillingness to continue attending the study for any reason and at any stage of the research, not obtaining the quorum score in Each of the selected subject tests for more than 2 times, failure to perform each of the selected subject tests, non-completion or incomplete completion of the satisfaction questionnaire. In coordination with the officials of the studied hospitals, a video guide for registering and entering the web application and participating in the training courses uploaded in PDF file format was provided to the nurses of the operating room through the WhatsApp messenger, and then they registered in the web application and participating in one of the training courses uploaded in the application, based on their needs and interests, and the progress process of the nurses in each of the training courses was followed through the results registered in the application and communication with each of them. The nurses in the study entered the application as described in phase 3.4 and participated in the uploaded training courses.

## Data collection tools

The three tools used in this study included the following:

### Tests to measure the level of knowledge of operating room nurses

The placement tests, the end of the basic level course, the end of the intermediate level course and the end of the advanced level course (final exam) in each of the educational topics, in which questions are designed by the teachers of each topic in the form of four-choice questions, and to evaluate the knowledge of operating room nurses. The budgeting of the questions for the placement and final exams was such that 25% of the questions were selected from basic level educational content, 50% from intermediate level content and 25% from advanced level content.

The number of questions and how to calculate the score in each topic are as follows:

#### Placement test

The heart anatomy’s exam consists of 40 four-choice questions and there is no negative score for wrong answers to the questions, and 0.5 points are awarded to the student for each correct answer. Therefore, the minimum and maximum score of the user is in the range of 0–20 and the duration of the test is 13 minutes. The way to interpret the obtained scores is that the learner with a score in the range of 0–7 is in the very poor level, 8–12 in the poor level, 13–17 in the average level, and 18–20 in the excellent level.

The test of the ergonomic principles in the operating room consists of 10 four-choice questions and there is no negative score for wrong answers to the questions, and 1 score is given to the students for each correct answer. Therefore, the minimum and maximum score of the user is in the range of 0–10 and the duration of the test is 4 minutes. The way to interpret the obtained scores is that the learner with a score in the range of 0–3 is in the very poor level, 4–5 in the poor level, 6–7 in the average level and 10–8 in the excellent level.

The test of the principles of moving, transferring and positioning the patient in the operating room consists of 15 four-choice questions and there is no negative score for wrong answers to the questions, and 1 score is given to the students for each correct answer. Therefore, the minimum and maximum score of the user is in the range of 0–15 and the duration of the test is 5 minutes. The way to interpret the obtained scores is that the learner with a score in the range of 0–7 is in the very poor level, 8–10 in the poor level, 11–12 in the average level, and 13–15 in the excellent level.

#### Basic level final exam

This test in the heart anatomy field consists of 19 four-choice questions and there is no negative score for wrong answers to the questions, and 1 score is given to the students for each correct answer. Therefore, the minimum and maximum score of the user is in the range of 0–19 and the duration of the test is 6 minutes. The passing score to enter the intermediate level course is equal to 13.

The test of the ergonomic principles in the operating room consists of 7 four-choice questions and there is no negative score for wrong answers to the questions, and 1 score is given to the student for each correct answer. Therefore, the minimum and maximum score of the user is in the range of 0–7 and the duration of the test is 2 minutes. The passing score to enter the intermediate level course is equal to 5.

The exam of the principles of moving, transferring and positioning the patient in the operating room consists of 10 four-choice questions and there is no negative score for wrong answers to the questions, and 1 score is given to the students for each correct answer. Therefore, the minimum and maximum score of the user is in the range of 0 to 10 and the duration of the test is 4 minutes. The passing score to enter the intermediate level course is equal to 7.

#### Intermediate level final exam

This test in heart anatomy field consists of 20 four-choice questions and there is no negative score for wrong answers to the questions, and 1 score is given to the students for each correct answer. Therefore, the minimum and maximum score of the user is in the range of 200 and the duration of the test is 7 minutes. The passing score to enter the advanced level course is equal to 14.

The exam of the ergonomic principles in the operating room consists of 10 four-choice questions and there is no negative score for wrong answers to the questions, and 1 score is given to the students for each correct answer. Therefore, the minimum and maximum score of the user is in the range of 0–10 and the duration of the test is 4 minutes. The passing score to enter the advanced level course is equal to 7.

The exam of the principles of moving, transferring and positioning the patient in the operating room consists of 10 four-choice questions and there is no negative score for wrong answers to the questions and 1 score is given to the participants for each correct answer, so the minimum and maximum score of the user is in the range of 0–10 and the duration of the exam is 4 minutes. The passing score to enter the advanced level course is equal to 7.

#### Final exam

This exam in the heart anatomy field consists of 40 four-choice questions and there is no negative score for wrong answers to the questions, and 0.5 points are awarded to the students for each correct answer. Therefore, the minimum and maximum score of the user is in the range of 0–20 and the duration of the test is 13 minutes. The passing score for the successful completion of the heart anatomy training course is equal to 15.

The exam of the ergonomic principles in the operating room consists of 10 four-choice questions and there is no negative score for wrong answers to the questions, and 1 score is given to the students for each correct answer. Therefore, the minimum and maximum score of the user is in the range of 0–10 and the duration of the test is 4 minutes. The passing score for the successful completion of the heart anatomy training course is equal to 6.

The test of the principles of moving, transferring and positioning the patient in the operating room consists of 15 four-choice questions and there is no negative score for wrong answers to the questions, and 1 score is given to the students for each correct answer. Therefore, the minimum and maximum score of the user is in the range of 0–15 and the duration of the test is 5 minutes. The passing score for the successful completion of the heart anatomy training course is equal to 11.

In order to determine the face validity of the questions of each test, Millman’s checklist and Blueprint were used and the results showed that the questions have appropriate face validity.

In order to evaluate the content validity of the questions of each of the tests of the training courses on the principles of patient movement, transfer and positioning in the operating room, heart anatomy and principles of ergonomics in the operating room, all the questions were given to 20 professors of medical sciences universities and only 10 of them examined the content validity of the questions using the Lausche index (CVR) and Waltz and Basel (CVI). The validity results indicated that the value of CVR and CVI for each of the tests in each of the mentioned training courses was 0.99 and 0.79, respectively, and the content validity of the instrument used in the present study was confirmed.

In order to measure the reliability of the questions of each of the tests using the Cronbach’s alpha method, a pilot study was conducted and during those tests, the questions of the placement tests, the basic level final exam, the intermediate level final exam and the final exam in each of the training courses of the principles of moving, transferring and positioning the patients in the operating room, heart anatomy and principles of ergonomics in the operating room were given to 30 operating room nurses and Cronbach’s alpha coefficient was calculated using SPSS version 16. The obtained results showed that the Cronbach’s alpha coefficient for placement tests, the basic level final exam, the intermediate level final exam and the final exam in the training course on the principles of moving, transferring and positioning of the patient in the operating room are equivalent to 0.812, 0.765, 0.805 and 0.819, respectively for each of the tests in the training course of heart anatomy, respectively, equivalent to 0.794, 0.780, 0.785, 0.760, and for each of the tests in the training course of ergonomic principles in the operating room, respectively, equivalent to 0.8, 0.749, 0.780, 0.was 826, which indicates the reliability of the questions.

### Demographic information questionnaire

A demographic information questionnaire was designed, which was used in the web application as a registration form for each learner, and in which questions such as place of employment hospital, the amount of work experience in the operating room, the field of study, the level of education, the specialized field of work, gender, user name and password are given.

### Satisfaction questionnaire

In order to measure the level of learner’s satisfaction with each of the training courses contents and the designed web application, a researcher-made questionnaire including 7 items with a 4-point Likert scale was used. The questionnaire’s interpretation was as follows: scores of 0–7, 8–14, 15–21, and 22–28 respectively mean lack of satisfaction, low satisfaction, moderate satisfaction, and high satisfaction of the learners with the contents of each of the educational courses, and the designed web application.

In order to determine the face and content validity of the satisfaction behavior questionnaire, the questionnaire was given to 20 professors of medical sciences universities and only 10 of them proceeded to determine the validity. The results of face validity indicated that all the items of the questionnaire had appropriate face validity in terms of the number of items, the comprehensibility of the sentences of each item and compliance with grammar. Also, the content validity of the items was evaluated using the Lausche Index (CVR) and Waltz and Basel (CVI) and the results indicated that the value of CVR and CVI for each test was 0.99 and 0.79, respectively, and the content validity of the questionnaire was confirmed.

In order to measure the reliability of the questionnaire using the Cronbach’s alpha method, a pilot study was conducted, during which the questionnaire was given to 30 operating room nurses, and the Cronbach’s alpha coefficient was calculated using SPSS version 16. The obtained results showed that Cronbach’s alpha coefficient was 0.794, which showed the reliability of the questionnaire.

## Statistical analysis

The collected data were analyzed using descriptive statistical tests (frequency and frequency percentage, mean and standard deviation), analytical tests (Paired sample t-test, independent samples t-test, ANOVA, Pearson correlation) and using SPSS software version 16.

## Results

After reviewing the data obtained from 36 operating room nurses, the data of 2 people were deleted due to not completing the satisfaction questionnaire and not participating in the final exam, and the data of 34 people (13 people in the course of the principles of movement, transfer and patient positioning in Operating room, 11 people in the course of heart anatomy and 10 people in the course of principles of ergonomics in the operating room) were analyzed.

The results of the demographic variables (Table 1) showed that among the nurses in the training group of the principles of patient movement, transfer and positioning in the operating room, 10 (76.9%) were women, 10 (76.9%) had a bachelor’s degree, 4 (30.7%) with work experience of 1–5 and 16–20 years, 7 people (53.8%) working in hospital one and 5 people (38.4%) working in general surgery field, 8 people (72.7%) in heart anatomy training group, 8 people (72.7%) have a bachelor’s degree, 5 people (45.4%) have 1–5 years of work experience, 7 people (63.7%) are working in 4 Hospital and 8 people (72.7%) are working in the field of cardiovascular surgery. And in the group of training course on principles of ergonomics in the operating room, 5 people (50%) are women, 6 people (60%) have a bachelor’s degree, 2 people (20%) have work experience 1–5, 6–10, 11–15, 20 16 and 21–25 years old, 5 people (50%) were working in hospital 1 and 2 people (20%) were working in the field of general surgery and endoscopy.

**Table 1:** Distribution of demographic information of operating room nurses participating in each of the specialized e-learning courses

Demographic information	Frequency (percent frequency)			*P*-value (chi-square)
	The group of principles of moving, transferring and positioning the patient in the operating room	The group of Cardiac anatomy group	The group of principles of ergonomics in the operating room	
**work experience**				*P* = 0.401
1–5	4(31)	5(46)	2(20)	
6–10	2(15)	4(36)	2(20)	
11–15	0(0)	0(0)	2(20)	
16–20	4(31)	1(9)	2(20)	
21–25	2(15)	1(9)	2(20)	
up 25	1(8)	0(0)	0(0)	
**Total**	13(100)	11(100)	10(100)	
**gender**				*P* = 0.051
Female	10(77)	3(27)	5(50)	
Man	3(23)	8(73)	5(50)	
**Total**	13(100)	11(100)	10(100)	
**Hospital of employment**				*P* = 0.004
One	7(54)	1(9)	5(50)	
two	4(31)	2(18)	4(40)	
three	2(15)	1(9)	1(10)	
four	0(0)	7(64)	0(0)	
**Total**	13(100)	11(100)	10(100)	
**Level of Education**				*P* = 0.452
Associate degree	3(23)	1(9)	3(30)	
Bachelor	10(77)	8(73)	6(60)	
Master	0(0)	2(18)	1(10)	
**Total**	13(100)	11(100)	10(100)	
**Field of specialized work surgery**				*P* = 0.012
Nerves	1(8)	0(0)	0(0)	
Orthopedics	3(23)	0(0)	1(10)	
General	5(37)	0(0)	2(20)	
Cardiovascular	1(8)	8(73)	0(0)	
Thorax	1(8)	3(27)	1(10)	
Urology	0(0)	0(0)	1(10)	
Plastic	0(0)	0(0)	1(10)	
jaw and face	0(0)	0(0)	1(10)	
Ear nose and throat	1(8)	0(0)	1(10)	
endoscopy	1(8)	0(0)	2(20)	
**Total**	13(100)	11(100)	10(100)	

Also, to investigate the relationship between demographic variables and educational courses, chi square test was used and the results showed that the variables of education level (*P* = 0.452), work experience (*P* = 0.401), gender (*P* = 0.051) and courses, no meaningful educational relationship was not seen; But there is a significant relationship between the hospital of the place of employment (*P* = 0.004) and the field of specialized surgery (*P* = 0.012) with training courses.

In order to compare the average knowledge scores of operating room nurses in general and also in two groups of training courses on principles of patient movement, transferring and positioning in the operating room, heart anatomy and principles of ergonomics in the operating room in the pre-intervention phase (level determination test) and in the stage after the intervention (final test), first the normality of the data distribution was checked using the Shapiro-Wilk test, according to the result of this test (*P* > 0.05) and the normality of the data distribution, in order to compare the scores, t-test was used and the results showed that the average knowledge scores of operating room nurses in general before and after the intervention were 5.91 ± 3.96 and 13.67 ± 3.77, respectively (*P*-value<0.001, mean difference: 7.76, 95%Cl: 6.62, 8.90) and significantly, the mean scores of knowledge in the stage after the intervention were higher than before the intervention (*P* < 0.001).(Table 2); Also, the average scores of nurses’ knowledge before and after the intervention in the course of the principles of moving, transferring and positioning the patient in the operating room are 6.07 ± 3.42 and 13.38 ± 1.32, respectively (*P*-value< 0.001, mean difference: 7.30, 95% CI: 5.15, 9.46), in the heart anatomy course 8.72 ± 3.97 and 18.18 ± 1.07 respectively (*P*-value< 0.001, mean difference: 9.45, 95%Cl: 6.98, 11.92) and in the course of principles of ergonomics in the operating room, respectively It was equivalent to 2.60 ± 1.57 and 9.10 ± 0.73, and significantly the mean scores after the intervention are higher than before the intervention (*P*-value< 0.001, mean difference: 6.50, 95% Cl: 5.53, 7.46) and significantly the mean scores of knowledge in the stage after the intervention was higher than before the intervention (*P* < 0.001) (Table 2).

**Table 2:** Comparison of the mean and standard deviation of knowledge scores of operating room nurses in three educational groups in the pre-and post-intervention phase

Educational group	Study stage	Mean ± Standard deviation	Lowest score	Highest score	Shapiro-Wilk	Paired sample t-test
**Principles of moving, transferring and positioning the patient in the operating room**	**Before the intervention (level determination test)**	6±3.42	1	12	*P*>0.05	*P*<0.001
	**After the intervention (final exam)**	13.3 ± 1.32	12	15		
**Anatomy of the heart**	**Before intervention**	8.7 ± 3.97	4	15	*P*>0.05	*P*<0.001
	**After intervention**	18.1 ± 1.07	16	20		
**Principles of ergonomics in the operating room**	**Before intervention**	2.6 ± 1.57	1	5	*P* > 0.05	*P* < 0.001
	**After intervention**	9.1 ± 0.73	8	10		
**Total**	**Before intervention**	5.9 ± 3.96	1	8	*P* > 0.05	*P* < 0.001
	**After intervention**	13.6 ± 3.77	15	20		

In order to determine the relationship between the average knowledge scores of operating room nurses in the training course groups of the principles of moving, transferring and positioning the patient in the operating room, heart anatomy and the principles of ergonomics in the operating room with demographic variables, Pearson, independent t and ANOVA tests were used. The results showed that there is a direct, weak and non-significant relationship between the average knowledge scores of operating room nurses in the training course group on the principles of moving, transferring and positioning patients in the operating room with the level of education (*r* = 0.165, *P* > 0.05). And there was an inverse, weak and non-significant relationship with work experience (*r* = 0.188, *P* > 0.05). Also, there is an inverse, strong and non-significant relationship between the knowledge scores of operating room nurses in the cardiac anatomy training group with the level of education (*r* = 0.547, *P* > 0.05) and a direct, strong and significant relationship with work experience (*r* = 0.622, *P* > 0.05). There is an inverse, strong and significant relationship between the average knowledge scores of operating room nurses in the training course group of principles of ergonomics in the operating room with the level of education (*r* = 0.667, *P* < 0.05) and a direct, strong and significant relationship with work experience (*r* = 0.707, *P* < 0.05).

There was no significant difference between the average knowledge scores of the nurses of the training course groups on the principles of movement, transferring and positioning of the patient in the operating room, heart anatomy and ergonomic principles in the operating room with the demographic variables of the hospital where they work, the field of specialized surgery and gender (*P*>0.05)).

Also, the results of the level of knowledge of the operating room nurses after performing the placement test showed that 8 (61.5%) of the nurses in the training group of the principles of patient movement, transferring and positioning in the operating room, 5 (45.5%) in the group of the heart anatomy training course) and 7 people (70%) of the nurses of the ergonomic principles training group in the operating room were at a very poor level (Table 3).

**Table 3:** Frequency distribution of the level of knowledge of operating room nurses in three educational groups at the end of the Placement test

Educational group	Level of knowledge	Frequency (percent)
**Principles of moving, transferring and positioning the patient in the operating room**	**very weak**	8(61.5)
	**weak**	3(23.1)
	**Medium**	3(15.4)
	**Total**	13(100)
**Anatomy of the heart**	**very weak**	5(45.5)
	**weak**	4(36.4)
	**Medium**	2(18.2)
	**Total**	11(100)
**Principles of ergonomics in the operating room**	**very weak**	7(70)
	**Weak**	3(30)
	**Medium**	0(0)
	**Total**	10(100)

The results of examining the amount and level of nurses’ satisfaction with the held electronic training courses showed that the average score of nurses’ satisfaction was 21.38 ± 5.83 and 22 (64.7) nurses were highly satisfied with the electronic training course; Also, the average score of nurses’ satisfaction in each of the training courses on the principles of moving, transferring and positioning the patient in the operating room, cardiac anatomy and principles of ergonomics in the operating room are 18.76 ± 7.15, 23.36 ± 3.82 and 4.88 ± 22.60 respectively, and 7 people (53.8%) from the nurses of the training course groups on the principles of patient movement, transferring and positioning in the operating room, 8 people (72.2%) from the heart anatomy group and 7 nurses (70%) from the ergonomic principles group in the operating room are highly satisfied (Table 4).

**Table 4:** The average satisfaction scores and satisfaction distribution of operating room nurses in each training course based on their needs using the web application

Educational group	Mean ± Standard deviation	Lowest score	Highest score	Satisfaction level	Frequency (percent)
**Principles of moving, transferring and positioning the patient in the operating room**	18.7 ± 7.15	8	27	**low**	3(23.1)
				**Medium**	3(23.1)
				**Hight**	7(53.8)
				**Total**	13(100)
**Anatomy of the heart**	23.3 ± 3.82	16	27	**Medium**	3(27.3)
				**Hight**	8(72.2)
				**Total**	11(100)
**Principles of ergonomics in the operating room**	22.6 ± 4.88	12	27	**Low**	1(10)
				**Medium**	2(20)
				**Hight**	7(70)
				**Total**	10(100)
**Total**	21.3 ± 5.83	8	27	**Low**	4(11.8)
				**medium**	8(23.5)
				**Hight**	22(64.7)
				**Total**	34(100)

## Discussion

This study sought to answer the question that to what extent electronic training on the specialized topics of the operating room profession based on a web application, leveled, asynchronous, personalized and based on the needs of nurses can lead to the improvement of specialized knowledge and nurses’ satisfaction. Is the operating room a new teaching method? The results showed that, in general, the knowledge of operating room nurses after the intervention (participation in electronic training courses, based on comprehensive and leveled needs) significantly improved compared to before the intervention, and the knowledge of nurses after the intervention. In each of the specialized electronic training courses, the principles of moving, transferring and positioning the patient in the operating room, heart anatomy and the principles of ergonomics in the operating room also increased significantly.

Today, one of the basic measures that leads to the efficiency of organizations is the creation or acquisition and continuous development of human resource through the implementation of training and improvement programs, which at the individual level increases the value of the individual, and at the organizational level, improves and develops the organization, and at the national and even transnational level, it leads to an increase in productivity [5, 6] and since official academic education and intensive and limited pre-service training are sufficient and acceptable, the hospital staff for Performing one’s duties in the hospital environment does not prepare one, the implementation of the educational program has become more necessary [7]. Due to the fact that each person has unique characteristics, the way of learning skills and learning needs are also different, so the first and most basic step in education is to examine the educational needs, which is a basis for preparation of special educational content is considered and provides a basis for setting goals and as a result, a suitable platform for organizing other important elements around prioritized needs; By preventing rework, it ultimately leads to increasing the effectiveness and efficiency of human resources, reducing waste, developing knowledge, skills, increasing job satisfaction and motivating employees [5, 8, 10–19]. In the study of Qalaei et al. (2013), the results showed that the lack of correct needs assessment has caused the lack of overlap between educational programs and the educational needs of nurses [7]. And for this reason, we initially examined the educational needs of operating room nurses and the results of this study showed that there is a need for training and improving knowledge in topics such as the principles of moving, transferring and positioning the patient in the operating room, heart anatomy and ergonomic principles in the operating room was more important than other topics.

The most widespread method of continuous training of medical personnel is the face-to-face training method, which many studies have shown that this traditional training method has many limitations, such as not recognizing the needs of learners and their personal differences, and not addressing high-level cognitive skills such as problem solving and creative thinking [21]; As a result, many researchers have emphasized that traditional educational methods need to be changed and modified by modern educational methods [21, 22]. Electronic education has been proposed as one of the complementary educational methods [21, 23, 26] and researchers have come to the conclusion that in order to correct the time and place limitations associated with traditional education, the possibility of lifelong learning and appropriate to the specific conditions of each learner, electronic education can increasingly provides the possibility of easy access to education and can be a suitable supplement for traditional education [21, 27, 43]; Therefore, in this study, in order to teach the topics required by operating room nurses, electronic education approaches were used in a leveled manner, and the results showed that the knowledge of operating room nurses participating in each of the electronic training courses on the principles of transferring, and patient positioning in the operating room, cardiac anatomy and principles of ergonomics in the operating room significantly increased compared to before participating in the courses (*P* < 0.001). The results of a review study by Rouleau and colleagues (2019) showed that the knowledge of nurses has increased, especially in the fields of calculation, preparation and prescription of medicine through electronic education [25]; Also, in a review study by Maertens and colleagues (2016), the results showed that the use of electronic education approach in surgical training has the same or more effectiveness than other educational methods in improving the knowledge of medical staff [44]; The results of bibani et al.’s study (2022) showed that the knowledge of nurses in the intervention group (using the e-learning approach) was significantly higher than the nurses in the control group (using face-to-face mock training method) (*P* < 0.05) [21] and the authors concluded that these results should encourage those responsible for continuing education to consider online education as a complementary and promising solution to ensure flexible continuing education sessions for health care personnel.

In the study of Sabbagh and colleagues (2017), the results showed that the knowledge level of nurses after the intervention (electronic training of patient safety principles) was significantly higher than before the intervention (*P* < 0.05) [79]; The results of the study by Sung et al. (2008) showed that the level of knowledge and satisfaction of nurses in the intervention group (combined teaching of pharmacology principles by electronic method and lectures) significantly improved compared to the control group (teaching principles of pharmacology by lecture method) (*P* < 0.05) [54] and these results show that blended learning by integrating e-learning and face-to-face classroom training is useful for increasing pharmaceutical knowledge. An e-learning program can reduce the lecture time and cost of repetitive topics such as medicine, which suggests that it can be an effective component in nursing education programs.

The results of the study by Hashemiparast et al. (2016) showed that the average knowledge score of the employees of the clinical departments of the selected hospitals of the universities of medical sciences in Tehran, Iran in the intervention group (teaching the principles of infection control electronically) was significantly higher than the control group. (*P* = 0.002) [80] and the authors concluded that despite the effectiveness of e-learning in learning and increasing learners’ awareness, the use of this method among health-related organizations requires empowering employees, removing barriers and infrastructures. The results of a review study by Feng and colleagues (2013) showed that electronic education leads to the improvement of learners’ knowledge [81] and the authors concluded that situational electronic education is an effective method to improve the performance of beginner learners. The effect of situational e-learning on improving cognitive ability is limited compared to traditional learning. Situational e-learning is a useful supplement to traditional learning for medical and nursing students. In the studies of Khatony and colleagues (2009) [82], Laine and colleagues (2019) [83], Gentizon and colleagues (2019) [24], Phaneuf and colleagues (2012), Vaona et al. (2018) [45], Bea et al. (2021) [46], Horiuchi et al. [84] Different types of medical care improve nurses’ knowledge compared to face-to-face training. Therefore, the web-based method is recommended as a complement to the face-to-face method for designing and presenting some topics of continuing education programs for nurses. The results of Van de Steeg et al.’s study (2015) showed that the average knowledge scores of nurses improved significantly after teaching the principles of delirium diagnosis in the elderly using an electronic method compared to before the intervention [85] and the authors believe this result, found that the e-learning course significantly improved nursing staff’s knowledge of delirium in all subgroups of participants and for all question categories. In contrast to other studies, the assessment of baseline knowledge showed that, overall, nursing staff were relatively knowledgeable about delirium. The results of the mentioned studies are consistent with the results of our study. Among the reasons for compatibility can be e-learning due to the use of different learning styles according to the needs and abilities of each learner and the adjustment of the learning speed by each learner according to his characteristics and to motivate learning due to the attractiveness of the educational environment. He pointed out that electronics leads to a better consolidation of learned material in the nurses’ memory and thus improves their knowledge [21, 22, 24, 44–53, 86–88].

Also, the results of the present study showed that the operating room nurses participating in each training course were delighted with the use of electronic and levelled education approaches.

Learners’ satisfaction is one of the essential factors in the effectiveness of new educational processes [32, 33, 43]. Nurses’ satisfaction with the education process leads to improved learning motivation and, as a result, improves their level of knowledge [43]. The results of Lhbibani et al.’s study [21] showed that the satisfaction of nurses in the intervention group (using the e-learning approach) was significantly higher than the nurses in the control group (using the face-to-face mock training method) (*P* < 0.05). Also, the results of Yazdannik et al.’s study [43] showed that the level of satisfaction of emergency department nurses in the intervention group (electronic patient triage training) was significantly higher than the nurses in the control group (face-to-face training) and authors concluded that using nursing professors’ electronic education programs can increase the level of satisfaction and motivation in the nursing mothers. Therefore, the use of this new educational method is recommended by managers and educational planners as an effective teaching. In the study of Costa and colleagues [89], the results showed that nurses were delighted with the electronic training on the principles of pain diagnosis in infants. The results of Narbona et al.’s study [90] showed that the nurses who participated in the electronic training course on evaluating patients’ pain intensity were delighted with the training course. The results of Chang et al.’s study [91] showed that the use of an e-learning approach in the in-service training of nurses resulted in 97.6% satisfaction of them. The results of Khoshnoodifar et al.’s study [92] showed that the level of nurse satisfaction with the cardiopulmonary resuscitation e-learning course was higher than that of the nurses in the control group (teaching the principles of cardiopulmonary resuscitation by lecture method) and authors concluded that satisfaction from CPR e-learning course was higher than those in nurses participating in the traditional training method. The results of the mentioned study are consistent with the results of our study. Among the compliance reasons, nurses can participate in training courses at any time and place based on their free time, exchange messages share their content and opinions with other learners in the online educational environment, and interact with course instructors [21, 41, 84, 86].

### Study implications

Based on the results of this study, it seems that the use of electronic education approach based on the needs of operating room nurses can be used as a complementary tool to conventional continuous education, and since this method allows interactive, personalized education, leveled, asynchronous and can be used at any time and place on a laptop, tablet and mobile phone, a wide range of operating room nurses in the hospitals of the Islamic Republic of Iran can use it for educational justice to many borders should be established in the country. However, there are studies to evaluate the generalizability and the effect of using the e-learning approach on the clinical skills of operating room nurses and to compare the effect of e-learning with other methods and educational tools on the knowledge and skills of the learners and the extent of consolidating the learned material in their memory.

### Strengths and limitations

Among the strengths of this study are the virtuality of training and evaluation of operating room nurses, training based on the needs of learners, organized training and appropriate to the knowledge level of each learner (personalized training), asynchronous training, the possibility of message exchange and interaction. Learners with each other and with instructors of training courses in the web application environment, using a new, low-cost training method, using leveled standardized tests made by researchers to measure the level of knowledge of operating room nurses in each of the training courses, online and It can be used at any place and time and with any smart device (laptop, tablet and mobile phone), reducing training costs, conducting a multicenter study and selecting subjects randomly, and among the limitations of the study, a small sample size and no control group can be named. Also, the low power of semi-experimental studies in generalizing the results obtained in the examined sample to the entire population compared to other studies, limited focus on null hypothesis tests and weak analytical samples are among the factors that threaten the validity of this study.

## Conclusion

The results of the present study showed that the use of an electronic education approach based on a web application, leveled, asynchronous, personalized and based on the needs of nurses led to the improvement of the knowledge of operating room nurses. Also, operating room nurses were highly satisfied with electronic training courses. It seems that e-learning can be used as a complementary educational tool and method for continuous training of operating room nurses in other specialized fields of operating room and surgery.

## Data Availability

The datasets used during the current study are available from the corresponding author upon reasonable request.
